# Disaturated-phosphatidylcholine and Surfactant protein-B turnover in human acute lung injury and in control patients

**DOI:** 10.1186/1465-9921-12-36

**Published:** 2011-03-24

**Authors:** Manuela Simonato, Aldo Baritussio, Carlo Ori, Luca Vedovelli, Sandra Rossi, Lorenza Dalla Massara, Sabina Rizzi, Virgilio P Carnielli, Paola E Cogo

**Affiliations:** 1 Department of Pediatrics, University of Padova, Padova, Italy; 2 Department of Medical and Surgical Sciences, University of Padova, Padova, Italy; 3 Department of Pharmacology, Anaesthesia and Critical Care, University of Padova, Padova, Italy; 4 Division of Neonatology, Department of Medical Sciences, Polytechnic University of Marche and Ospedali Riuniti of Ancona, Ancona, Italy

## Abstract

**Background:**

Patients with Adult Respiratory Distress Syndrome (ARDS) and Acute Lung Injury (ALI) have low concentrations of disaturated-phosphatidylcholine and surfactant protein-B in bronchoalveolar lavage fluid. No information is available on their turnover.

**Objectives:**

To analyze disaturated-phosphatidylcholine and surfactant protein-B turnover in patients with ARDS/ALI and in human adults with normal lungs (controls).

**Methods:**

^2^H_2_O as precursor of disaturated-phosphatidylcholine-palmitate and 1^13^C-Leucine as precursor of surfactant protein-B were administered intravenously to 12 patients with ARDS/ALI and to 8 controls. Disaturated-phosphatidylcholine and surfactant protein-B were isolated from serial tracheal aspirates, and their fractional synthetic rate was derived from the ^2^H and ^13^C enrichment curves, obtained by gas chromatography mass spectrometry. Disaturated-phosphatidylcholine, surfactant protein-B, and protein concentrations in tracheal aspirates were also measured.

**Results:**

1) Surfactant protein-B turned over at faster rate than disaturated-phosphatidylcholine both in ARDS/ALI patients and in controls. 2) In patients with ARDS/ALI the fractional synthesis rate of disaturated-phosphatidylcholine was 3.1 times higher than in controls (p < 0.01), while the fractional synthesis rate of surfactant protein-B was not different. 3) In ARDS/ALI patients the concentrations of disaturated-phosphatidylcholine and surfactant protein-B in tracheal aspirates were markedly and significantly reduced (17% and 40% of the control values respectively).

**Conclusions:**

1) Disaturated-phosphatidylcholine and surfactant protein-B have a different turnover both in healthy and diseased lungs. 2) In ARDS/ALI the synthesis of these two surfactant components may be differently regulated.

## Background

Adult Respiratory Distress Syndrome (ARDS) is characterized by bilateral radiographic infiltrates and a ratio of the partial pressure of arterial oxygen to the fraction of inspired oxygen (PaO_2_/FiO_2_) <200. Acute Lung Injury (ALI) is a less severe form of respiratory distress with a PaO_2_/FiO_2 _between 200 and 300 [1]. In both clinical entities reduced lung compliance and surfactant inactivation contributes to the respiratory failure [2-4]. Proposed mechanisms of lung injury include destruction of the air-water interface by alveolar edema, phospholipid degradation by secretory phospholipases, degradation of surfactant proteins by proteases [5,6], damage by reactive oxygen species and decreased synthesis of surfactant components by damaged type II cells [7]. Moreover, leakage of plasma proteins into the alveoli is thought to decrease alveolar stability [8]. In vitro, proteins have been shown to compete with the lipids for adsorption at the air water interface, leading to the formation of an interfacial film less prone to incorporate surfactant phospholipids [9].

Pulmonary surfactant is composed by 90% lipids, mostly phospholipids, and 10% of specific proteins [10]. Surfactant Protein B (SP-B) is a small hydrophobic protein synthesized as a preprotein (pro-SP-B) and processed to mature form en route from the Golgi complex to the lamellar bodies [11]. Mature SP-B is secreted as a surfactant component into the alveolar space where it is mainly found as a dimer [12]. Clara cells also express pro-SP-B, but they do not process the protein to the mature form [13]. SP-B enhances the rate of phospholipid adsorption to the alveolar air/water interface [14], decreases surface tension by interfering with the attractive forces acting between water molecules [15] and has anti-inflammatory properties [2,3].

Clinical studies have shown that disaturated-phosphatidylcholine (DSPC) is markedly decreased in bronchoalveolar lavage fluid of adult ARDS patients, whereas the phosphatidylcholine content is only slightly decreased [2,3]. SP-B has also been reported to be reduced by 25-55% in tracheal aspirates of adult ARDS/ALI patients [2,6] but in association with normal levels of SP-B mRNA [16]. Conversely a recent study performed in children confirmed a significant decrease of DSPC but no significant changes in SP-B levels in bronchoalveolar lavage fluid of ALI/ARDS children compared with controls[17]. Decreased levels of DSPC and SP-B could be due to increased degradation, decreased synthesis or both. The goal of this investigation was to measure the rate of synthesis of DSPC and SP-B in ARDS/ALI patients and in controls with normal lungs.

## Methods

Patients were studied from 2003 to 2006. ARDS and ALI cases were defined by the AECC criteria [1]. Twelve patients were recruited within 72 hours from the onset of the respiratory failure. Causes of ARDS/ALI were pneumonia in 6 patients, polytrauma in 4, pancreatitis in 1 and aspiration of gastric contents in 1. Eight patients without lung disease, who needed mechanical ventilation after major surgery or for neurological failure, were enrolled as controls. Exclusion criteria were: presence of liver failure, defined as transaminases (GOT, GPT) >3 times normal values, renal failure (creatinine >2 times normal values), burns >30% body surface area, bone marrow or lung transplantation. The local ethical committee approved the study, and informed consent was obtained for all the patients.

Clinical data, ventilator parameters, arterial blood gas analysis and trascutaneous saturation were recordered at the start of the study and then every 6 hours during the study period. Routine blood tests performed at the admission to the ICU and during the study according to the standard unit practice were also recorded in order to detect signs of nosocomial infection or of organ failure. For each patient SAPSII score was calculated at study entry.

### Methods and study design

All patients received a constant intravenous infusion of 1 g 1-^13^C Leucine (Cambridge Isotope Laboratories, Andover, MA) dissolved in saline for 24 h. ^2^H_2_O (Cambridge Isotope Laboratories, Andover, MA) was administered as a 25 ml bolus at the study start and then, every 12 hours over the next 36 hours, as intermittent boluses corresponding to 0.0625% of fluid intake, to maintain steady state of deuterium enrichment in body water [18].

Blood (0.6 ml), urine (1 ml) and tracheal aspirates [19] were collected at times 0, 6, 12, 18, 24, 30, 36, 42, 48 hours and then every 12 hours until extubation. Tracheal aspirates and blood samples were centrifuged at 400 g and 1300 g respectively and supernatants were stored at -80°C.

### Tracheal aspirates protein and phopholipid analysis

Total proteins from tracheal aspirates were determined at time 0 or within the first 24 hours [20]. Phospholipid phosphorous was measured in tracheal aspirate samples till 48 hours [21] from the start of the study.

DSPC was extracted and separated from tracheal aspirates according to standard methods [22] and DSPC fatty acids derivatized as methyl-esters and measured by gas chromatography [19].

SP-B amounts were quantified by ELISA on unfractionated tracheal aspirates obtained at the beginning of the study or within 24 hours [23] using a rabbit antiserum directed against mature SP-B [24].

DSPC, SP-B and protein concentrations in aspirates, were normalized to alveolar lining fluid using the urea method (BioAssay Systems, Hayward, CA) [25].

### Isolation of SP-B from tracheal aspirates

SP-B was isolated from tracheal aspirates and hydrolyzed as previously published [26]. Briefly, SP-B was isolated from lipid extract with Bond Elute NH_2 _containing 100 mg resin (Supelco, Milano, Italy) preconditioned with 3-5 ml of chloroform. After loading the sample, the columns were eluted sequentially with 3 ml of the following chloroform/methanol/acetic acid mixtures: 20:1:0; 9:1:0; 4:1:0; 4:1:0.025; 3:2:0; 1:4:0; 1:9:0. SP-B fraction (4:1:0 and 4:1:0.025) were pooled together, dried under nitrogen and hydrolysed to free amino acids by 0.5 ml of HCl 6 N for 24 h at 110°C. Individual amino acids were derivatized into their N-acetyl-n-propyl derivatives [27] and the ^13^C enrichment of leucine measured by mass spectrometry (GC-MS Voyager, Thermoquest, Milan, Italy).

### Plasma leucine enrichment

One hundred μl of plasma were deproteinized with sulphosalicilic acid (6% w/v) and plasma amino acids were derivatized according to Husek [28]. Free plasma leucine enrichment was measured by gas chromatography mass spectrometry. Results were expressed as Mole Percent Excess with reference to a calibration curve for 1-^13^C Leucine.

### Urine deuterium enrichment

One hundred μl of urine were deproteinized with sulphosalicilic acid (6% w/v) and diluted 1:10 with local distilled reference water. Urine deuterium enrichment was analysed by a High Temperature Conversion Elemental Analyser coupled with an Isotope Ratio-Mass Spectrometer (TC-EA-IRMS, Thermo Scientific, Bremen, Germany) [29]. Enrichment was expressed as delta ‰.

The ^2^H enrichment of DSPC-palmitate was analyzed by gas chromatography-IRMS and expressed in delta ‰ after correction for isotopic contribution of the derivative group [18].

### Calculations

All kinetic measurements were performed assuming a steady state. The assumption was based on the following considerations: 1) in all patients plasma 1^13^C leucine and urine ^2^H_2_O enrichments reached steady state within 6 h from the start of the isotope infusion; 2) the slope of the enrichment curve over time did not deviate significantly from zero between time 6 h and time 24 h for plasma leucine and between time 6 h and time 36 h for urine ^2^H_2_O; 3) the concentrations of DSPC and SP-B in tracheal aspirates did not change significantly during the first 48 hours of the study.

Tissue-bound and alveolar surfactant was regarded as one pool. The fractional synthesis rate was calculated as previously reported [18,19].

Clinical and kinetic variables were expressed as mean ± SD or median (interquartile range) as appropriate. Statistical analysis was performed by a non-parametric test (Mann-Whitney test). Significance was defined as p < 0.05. Data were analysed using the statistical package SPSS 15 (SPSS Inc, Chicago, IL).

## Results

### Clinical characteristics

Twelve ARDS/ALI and 8 control adults were studied. Clinical characteristics and ventilator parameters are reported in Table 1. Only 4 of the 8 controls were on mechanical ventilation, the remaining patients were on spontaneous breathing through a tracheostomy. All ARDS/ALI patients exhibited severe derangement of gas exchange at the time of the study start (which corresponded to the first tracheal aspirate collection) with a PaO_2_/FiO_2 _ratio of 185 ± 61. Thereafter PaO_2_/FiO_2 _values progressively improved, but remained markedly lower than control values (Table 1). Two of the 20 patients (both with ARDS) died within 30 days from the study start.

**Table 1:** Clinical characteristics and ventilator parameters of study patients.

VARIABLE	PATIENTS		p
		
	ARDS/ALI (N = 11)	CONTROL (N = 8)	
Male/Female (n°)	6/5	4/4	

Age (y)	53 ± 16	48 ± 24	0.66

Body weight (Kg)	80 ± 16	69 ± 19	0.21

Survival	82%	100%	

SAPS II	37 ± 12	37 ± 5	0.46

PIP at the start of the study (cm H_2_O)	25 ± 6	14 ± 4 (4/8)	<0.01

PIP during the study (cm H_2_O)	24 ± 6	14 ± 4 (4/8)	0.05

PEEP at the start of the study (cm H_2_O)	9 ± 2	3 ± 1 (4/8)	<0.01

PEEP during the study (cm H_2_O)	9 ± 2	3 ± 0.4 (4/8)	<0.01

PaO_2_/FiO_2 _at the start of the study	185 ± 61	365 ± 72	<0.001

PaO_2_/FiO_2 _during the study period	208 ± 46	383 ± 82	<0.001

AaDO_2 _at the start of the study (mmHg)	309 ± 111	71 ± 27	<0.001

AaDO_2 _during the study (mmHg)	255 ± 84	74 ± 41	<0.001

Tidal Volume (ml/Kg bw)	7 ± 1	7 ± 1 (4/8)	0.54

Clinical characteristics and ventilatory parameters of study patients. Mean ± SD. PaO_2_/FiO_2 _= mean oxygen tension in arterial blood/inspiratory oxygen fraction. PEEP = positive end expiratory pressure. PIP = peak inspiratory pressure. AaDO_2 _= Alveolar arterial oxygen gradient. APACHE II = scores on the acute physiology and chronic health evaluation. Values compared with Mann-Whitney test.

### Surfactant composition

Total proteins, total phospholipids, DSPC and SP-B concentrations, normalized to epithelial lining fluid, at study start are shown in Table 2. In patients with ARDS/ALI total protein concentration were higher, (p < 0.05), total phospholipids were not different, and DSPC and SP-B were markedly and significantly lower than in controls. DSPC and SP-B concentrations were 16.9% and 40.5% of the control values respectively.

**Table 2:** Surfactant composition of tracheal aspirates

VARIABLE	PATIENTS		p
		
	ARDS/ALI	CONTROLS	
Total phospholipids (mg/ml ELF)	0.98 (0.57-1.30)	2.30 (1.03-1.66)	0.07

Total protein (mg/ml ELF)	68.6 (46.3-97.2)	44.7 (33.7-49.1)	<0.05

PPQ	0.010 (0.014-0.036)	0.034 (0.024-0.051)	<0.01

SP-B (μg/ml ELF)	1.7 (0.8-2.9)	4.2 (3.4-5.8)	<0.05

SP-B (%PL)	0.21 (0.08-0.36)	0.21 (0.11-0.44)	0.7

DSPC (mg/ml ELF)	0.11 (0.08-0.24)	0.65 (0.50-2.59)	<0.01

Mean DSPC (%PL)	50 (43-51)	54 (52-58)	<0.01

All data represent as median and interquartile range. PPQ = Phospholipid-to-Protein quotient. ELF = epithelial lining fluid. Total protein, total phospholipids, DSPC and SP-B were determined in one tracheal aspirates at the start of the study or within 24 h. Values were corrected for dilution using the urea method. DSPC (%PL) was determined in all tracheal aspirates collected till the end of the study. Values were compared using Mann-Whitney test.

DSPC, as percentage of total phospholipids, was lower in ARDS/ALI than in controls throughout the study (p < 0.01).

### Surfactant DSPC and SP-B kinetics

**Figure 1:**
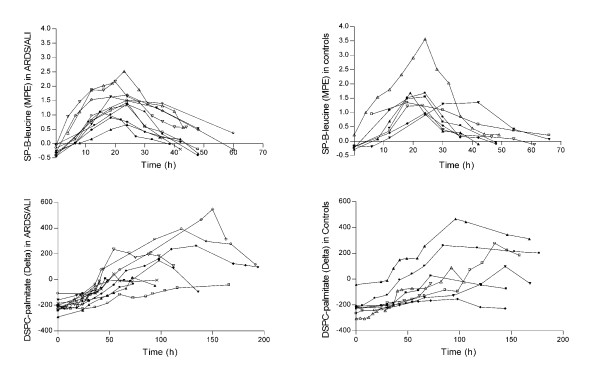
**SP-B leucine and DSPC-palmitate enrichment curves in ARDS/ALI and in control patients**. The ^13^C-leucine (top panels) and deuterium-palmitate (bottom panels) enrichment curves of SP-B and DSPC obtained from tracheal aspirates. Each symbol represents a different subject. MPE: mole percent excess; Delta (^2^H/^1^H vs smow).

DSPC concentration in tracheal aspirates was measured every 6 hours for the first 48 hours in all ARDS/ALI patients to assess the steady state of the surfactant pool. Except for 1 ARDS patient, who was excluded from the study for an increasing DSPC concentration over time, all patients had DSPC concentration within 10% of coefficient of variation (data not shown), suggesting a steady state condition during the time frame of kinetics measurement. Enrichment curves of DSPC-palmitate and SP-B-leucine from ARDS patients and control subjects are shown in Figure 1. In most subjects SP-B enrichments returned to baseline within 60 hours from the start of the tracer infusion, while DSPC enrichments took about 150-200 hours to return to baseline.

SP-B turned over at a much faster rate than DSPC both in controls and in patients with ARDS/ALI (Figure 1). In controls SP-B turned over at a rate 28 times faster than DSPC.

**Figure 2:**
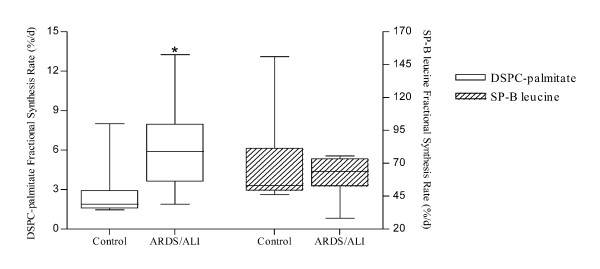
**Fractional synthetic rate of DSPC-palmitate and SP-B-leucine in ARDS/ALI and control patients**. Synthesis of DSPC-palmitate results to be significantly higher in ARDS/ALI group. SP-B-leucine synthesis is similar in the two groups. Results are expressed as median and interquartile ranges. * Significantly different from controls (p < 0.01 by Mann-Whitney test).

In ALI/ARDS the fractional synthesis rate of DSPC was 3.1 times higher than in controls (p < 0.01), while the fractional synthesis rate of SP-B was not statistically different than in control subjects (Figure 2).

## Discussion

This paper provides novel information on the turnover of DSPC and SP-B in normal subjects and in patients with ARDS/ALI, while confirming that patients with ARDS/ALI have decreased levels of DSPC and SP-B and increased protein concentration in tracheal aspirates [2,4-6,16,30,31].

In agreement with previous animal studies [24,32], we found that SP-B is turned over at a faster rate than DSPC both in controls and in ARDS/ALI. Since SP-B and DSPC are packaged into lamellar bodies and then are secreted together, their different turnover rate could be explained by partitioning into distinctive alveolar structures (i.e. DSPC could distribute preferentially to the interfacial film or stay there for a longer time), by an intrinsically faster turnover of SP-B in the alveolar space or by the use of different pathways of recycling before packaging into lamellar body membranes. Human studies do not allow to further discriminate among these possibilities, since lung tissue could not be sampled. From animal experiments, though, we can extrapolate that all these mechanisms could be at play [24,32].

In the present study we used ^2^H_2_O as metabolic precursor for DSPC synthesis. This is at variance from other studies by our group where we used other metabolic precursors, such as deuterated palmitate or uniformely labeled ^13^C glucose [18,33,34]. We chose the heavy water for its ease to use and because in preterm and term infants we found that heavy water gives estimates of DSPC synthesis similar to those obtained using other metabolic precursors [34]. ARDS/ALI patients showed an increased synthesis rate of DSPC but not of SP-B. If the increased synthesis rate of DSPC seems a logical compensatory mechanism to increased phospholipid degradation or monolayer inactivation in the injured lungs, the finding that in ARDS/ALI the rate of synthesis of SP-B is comparable to control subjects, is unexpected. Possible explanations are that ARDS/ALI patients cannot mount a compensatory enhanced synthesis of SP-B or conversely that the decrease of airway SP-B in our ARDS patients was not big enough to elicit a homeostatic response. In agreement with the last interpretation, a recent study with a transgenic mouse model expressing human TNF-α in respiratory epithelial cells showed that, after a 36-61% decrease of airway SP-B, the level of SP-B mRNA in lung tissue remained unchanged [16].

Differently from SP-B, the lung appeared to "sense" the larger decrease in airway DSPC content and increased three fold its fractional rate of synthesis. The mechanism underlying this response remains unclear. A recent article investigating the role of signal transducer and activator of transcription-3 (STAT-3), which is activated by members of IL-6-like group of proinflammatory cytokines [35], found that in mice treated with LPS the synthesis of DSPC and SP-B increased in a STAT-3 dependent way [36].

In this study we have measured also DSPC and SP-B concentration in epithelial lining fluid as a proxy of surfactant pool. The estimation of airway surfactant pool by the measurement of individual surfactant components obtained from tracheal aspirates has limitations, since the recovery may vary, in spite of the standardization of the suction technique and the correction for dilution. Therefore the low concentrations of DSPC and SP-B recovered from tracheal aspirates of patients with ARDS/ALI could be due either to decreased surfactant concentration in the airways, or to the fact that collapsed areas may be less prone to release surfactant recoverable by aspiration or even to the sampling technique that could be affected by clinical severity. These observations can partially explain the controversial results reported on surfactant SP-B amount during ARDS/ALI [2,6,17].

Kinetic data obtained from tracer studies have the advantage of being independent from recovery. Tracer studies were classically performed in animals using radioactive tracers, and they showed that surfactant specific proteins A, B and C were turned over faster than DSPC [24,32]. Using stable isotopes we can now perform kinetic studies in humans and even in preterm infants. By this approach we previously measured the rate of synthesis of DSPC and SP-B in newborn infants with and without respiratory failure [18,26]. Furthermore, by administering DSPC labelled with stable isotopes through the airways, we showed that in a population of patients with ARDS the airway DSPC pool was 10 fold smaller than in controls [37].

A further limit on the estimation of the airway surfactant composition and pool by tracheal aspirates derives from the fact that the currently available ELISA methods to measure SP-B cannot distinguish between the mature protein and its pro-forms, nor do they recognize modified SP-B, like oxidized SP-B, whose possible pathogenetic role has been recently underlined [38]. In this paper we separated mature SP-B by sorbent chromatography and thus provides kinetic data regarding solely mature SP-B.

Since the concentrations of DSPC and SP-B in the tracheal aspirates of patients with ARDS/ALI did not change significantly during the sampling period, we assumed that patients were at steady state, with synthesis matching degradation for both surfactant components. However, due to the uncertainty about the true dimensions of the alveolar pool of DSPC and SP-B, the present data did not allow us to calculate the absolute rate of synthesis of these compounds [39].

In conclusion our paper provides original data on DSPC and SP-B turnover "*in vivo*" in humans. Our results can be summarized as follows: 1) DSPC and SP-B turned over with different kinetics in the airways both in human adults with normal lungs and in patients with ARDS/ALI, 2) in patients with ARDS/ALI in spite of the low concentration of DSPC and SP-B in tracheal aspirates, the fractional synthesis rate of DSPC increased, while that of SP-B did not change significantly.

## Competing interests

The authors declare that they have no competing interests.

## Authors' contributions

MS and LV performed all the experiment, MS also calculated the data and wrote in part the manuscript. AB has been involved in revising critically the manuscript and in data interpretation. PEC contributed to experimental design, data interpretation and in drafting the manuscript. CO and VPC participated in the design of the study and help revising the manuscript. LDM has made substantial contribution to the acquisition of data and to the recruitment of patients. SR and SR contributed to the revision of the manuscript and to the recruitment of patients. All authors read and approved the final manuscript.
